# A surveillance summary of smoking and review of tobacco control in Jordan

**DOI:** 10.1186/1744-8603-5-18

**Published:** 2009-12-01

**Authors:** Adel Belbeisi, Mohannad Al Nsour, Anwar Batieha, David W Brown, Henry T Walke

**Affiliations:** 1 Ministry of Health, Amman, Jordan; 2 Consultant to Centers for Disease Control and Prevention, Atlanta, Georgia, USA; 3 Jordan University of Science and Technology, Irbid, Jordan; 4 Centers for Disease Control and Prevention, Atlanta, Georgia, USA

## Abstract

The burden of smoking-related diseases in Jordan is increasingly evident. During 2006, chronic, noncommunicable diseases (NCDs) accounted for more than 50% of all deaths in Jordan. With this evidence in hand, we highlight the prevalence of smoking in Jordan among youth and adults and briefly review legislation that governs tobacco control in Jordan. The prevalence of smoking in Jordan remains unacceptably high with smoking and use of tobacco prevalences ranging from 15% to 30% among students aged 13-15 years and a current smoking prevalence near 50% among men. Opportunities exist to further reduce smoking among both youth and adults; however, combating tobacco use in Jordan will require partnerships and long-term commitments between both private and public institutions as well as within local communities.

## Findings

The negative health consequences of smoking and second hand smoke exposure are well documented [1-3]. The World Health Organization (WHO) estimates that there are more than one billion current smokers worldwide and that more than 80% of the world's smokers live in low- and middle-income countries [1]. An estimated 5.4 million people die from diseases directly related to cigarette smoking worldwide each year [1] and millions more are affected by the nonfatal consequences of tobacco use. Unabated, tobacco-related deaths are estimated to increase to more than eight million a year by 2030, and 80% of those deaths will occur in the developing world [1].

The burden of smoking-related diseases in Jordan is increasingly evident [4-6]. During 2006, chronic, noncommunicable diseases (NCDs) accounted for more than 50% of all deaths in Jordan [7]. Deaths from heart disease and stroke (ICD-10 codes I00-I99) accounted for a third of all deaths, and malignant neoplasms (C00-C97) were responsible for about 13% of deaths, with lung cancer being the leading cause of cancer death. Nearly 60% of deaths from malignant neoplasms occurred among people younger than 65 years, and approximately one-third of those who died from heart disease and stroke were aged 65 or younger. Moreover, the economic consequences of smoking-related morbidity and mortality are profound [1]. In addition, according to national estimates, smokers in Jordan spend an estimated JD 250 million annually on tobacco products [8]. With this evidence in hand, we provide an update of the prevalence of smoking in Jordan among youth and adults. Because legislation is central to effective tobacco control [9], we briefly review legislation that governs tobacco control in Jordan.

For this report, data were derived from national health surveys conducted by the Jordan Ministry of Health (MOH) as well as surveys conducted by the MOH in collaboration with the WHO and the United States Centers for Disease Control and Prevention (CDC).

### Smoking among Youth

The prevalence of tobacco smoking among youth was obtained from two sources, the Global Youth Tobacco Survey (GYTS) and the Global School-based Student Health Survey (GSHS). The GYTS, conducted in Jordan during 1999, is a school-based survey of students aged 13-15 years in public or private schools. The GSHS, also a self-administered, school-based survey conducted primarily among students 13-15 years of age, was conducted in Jordan during 2004 and 2007.

Both surveys employ a multistage sample design with schools selected proportional to enrollment size and classrooms chosen randomly within selected schools. All students in selected classes are eligible for participation, and surveys can be administered during one regular class period. During 1999, a total of 3912 students participated in the Jordan GYTS with an overall response rate of 83.9% [10]. A detailed description of the GTYS and its methodology is provided elsewhere [11]. For the 2004 Jordan GSHS, 2457 questionnaires were completed in 26 schools with an overall response rate of 95%. For the 2007 Jordan GSHS, 2197 questionnaires were completed in 25 schools with an overall response rate of 99.8%. Further details of the GSHS can be obtained at http://www.who.int/chp/gshs and http://www.cdc.gov/gshs.

The estimated prevalence of ever smoking among youth is shown in Tables 1 and 2. Current smoking prevalence ranged from 18% in 1999 to about 13% in 2004 and 16% in 2007. The prevalence of current smoking was substantially greater among boys than girls, with approximately 1 in 5 boys reporting that they currently smoke compared to 7 to 10% of girls. Use of other forms of tobacco was also high among both boys and girls. Nearly 1 in 3 boys reported current use of other forms of tobacco during 2007 and roughly 17% of girls reporting current use of other forms.

**Table 1:** Prevalence of ever smoking, current smoking and current tobacco use among youth (aged 13-15 years) in Jordan, Global Youth Tobacco Survey (GYTS), 1999

	Boys	Girls	Overall
Ever smoked cigarettes	44.1%	25.8%	36.4%
Currently smoke cigarettes	22.6%	11.4%	18.3%
Currently use any form of tobacco	27.5%	15.2%	22.9%

* GYTS sample size, 3912

Source: GYTS data obtained online from: http://www.cdc.gov/tobacco/global/GYTS/factsheets/emr/1999/jordan_factsheet.htm Accessed 11 June 2009.

Notes. Lifetime prevalence of smoking was obtained from an affirmative response to the question, "Have you ever tried or experimented with cigarette smoking, even one or two puffs?". Youth were also asked the question "During the past 30 days (one month), on how many days did you smoke cigarettes?". Those who responded one ore more days were considered current smokers. Similarly, youth were asked about use of smoked tobacco products other than cigarettes (e.g. cigars, water pipe, cigarillos, little cigars, pipe) and use of any form of smokeless tobacco products (e.g. chewing tobacco, snuff, dip) during the previous 30 days. Those responding affirmatively were considered to currently use other forms of tobacco.

**Table 2:** Prevalence of current smoking and current tobacco use on one or more days during the 30 days preceding the survey among youth (aged 13-15 years) in Jordan, Global School-based Student Health Survey (GSHS), 2004 and 2007

	2004	2007
	(n = 2457)	(n = 2197)
Smoked cigarettes on one or more days during the 30 days preceding the survey		
Boys	19.2% (14.9-23.5)	22.7% (18.1-27.2)
Girls	6.6% (3.8-9.4)	8.7% (6.1-11.2)
Overall	12.6% (10.1-15.1)	15.6% (11.0-20.2)
Used any form of tobacco on one or more days during the 30 days preceding the survey		
Boys	28.4% (25.5-31.3)	33.5% (29.2-37.9)
Girls	12.2% (9.9-14.5)	16.5% (11.6-21.5)
Overall	19.9% (17.7-22.1)	24.9% (19.4-30.3)

95% confidence interval reported in parentheses

Source: GSHS data obtained online from http://www.who.int/chp/gshs/jordan/en Accessed 11 June 2009.

Notes. As part of the survey, youth are asked the number of days during the 30 days preceding the survey that they smoked cigarettes. Those reporting that they smoked cigarettes on one or more days were considered current smokers. Similarly, youth were asked the number of days they used any other form of tobacco during the 30 days preceding the survey.

### Smoking among Adults

The prevalence of tobacco smoking among adults was obtained from behavioral risk factor surveys (BRFS) conducted by the Jordan MOH during 2002, 2004 and 2007. A detailed description of the Jordan BRFS is provided elsewhere [4,5,12]. Briefly, during 2002 questions about behavioral risk factors and NCD prevalence were added to the Jordan Department of Statistics' quarterly, multistage, cross-sectional employment and unemployment survey. During 2004 and 2007, the Jordan MOH conducted its second and third BRFS, respectively, among a nationally representative sample of adults aged ≥ 18 years. Similar to 2002, a multistage sampling design was used to select households using the master sampling frame of census enumeration blocks from the 2004 Jordan census to select the sample of blocks, or primary sampling areas, from which households were selected. In each household, one adult aged 18 years or older was randomly selected and interviewed in person in Arabic. During 2004, a total of 3520 households were selected and 3334 adults were interviewed; a response rate of 94.7%. During 2007, a total of 3688 households were selected and 3654 adults were successfully interviewed; a response rate of 99.1%. Smokers were classified as "ever smokers" (i.e., smokers who had smoked ≥ 100 cigarettes during their lifetime) or "current smokers" (i.e., smokers who had ever smoked 100 cigarettes and currently smoke every day or some days).

**Figure 1:**
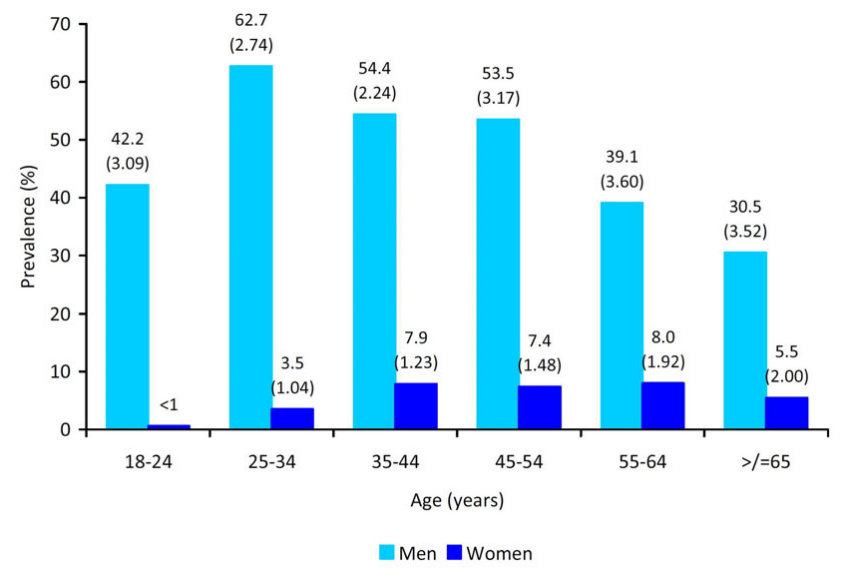
**Age-specific current smoking prevalence among adults aged 18 years or older by gender, Behavioral Risk Factor Surveillance System, Jordan, 2007**.

**Figure 2:**
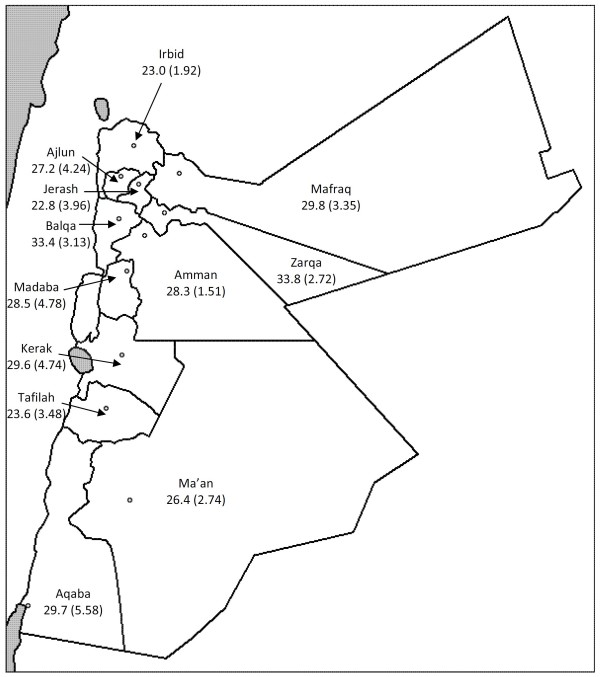
**Age-standardized current smoking prevalence among adults aged 18 years or older by governorate, Behavioral Risk Factor Surveillance System, Jordan, 2007**.

During, 2007, nearly 40% of all adults aged 25 years or older reported having smoked at least 100 cigarettes during their lifetime (Table 2). Overall during 2007, the age-standardized prevalence of current smoking was 28% (standard error [SE], 0.86) with nearly half of men reporting current smoking behaviour compared to 5% of women (Table 3). Men aged 25-34 years had the highest (63%) prevalence of current smoking and women aged 18-24 years had the lowest (<1%) prevalence (Figure 1). By governorate in 2007, the age-standardized prevalence of current smoking ranged from 23% in Irbid and Tafela to 33% in Balqa and Zarqa (Figure 2).

**Table 3:** Survey participant characteristics and age-specific and age-standardized smoking prevalences among adults aged 18 years or older by participant characteristics, Behavioral Risk Factor Surveillance System, Jordan, 2007

Characteristic	Survey Participant Characteristics n = 3654 % (SE)	Prevalence of Lifetime Smoking n = 1409 % (SE)	Prevalence of Current Smoking n = 1080 % (SE)
**Age, yrs**			
18-24	14.9 (0.64)	25.0 (1.96)	23.4 (1.95)
25-34	19.6 (0.76)	41.0 (2.13)	37.2 (2.06)
35-44	26.7 (0.80)	40.7 (1.60)	32.9 (1.57)
45-54	15.4 (0.62)	38.6 (2.16)	28.5 (2.02)
55-64	12.7 (0.58)	39.6 (2.61)	23.6 (2.29)
≥65	10.8 (0.58)	40.3 (2.97)	19.4 (2.39)
Gender*			
Men	53.1 (0.87)	61.8 (1.21)	48.2 (1.27)
Women	46.9 (0.87)	7.8 (0.67)	5.1 (0.54)
Education*			
Never attended school	11.4 (0.58)	24.7 (4.98)	18.6 (4.83)
Primary school	32.0 (0.87)	44.1 (1.74)	35.3 (1.90)
Secondary or technical school^a^	42.7 (0.87)	36.5 (1.56)	26.8 (1.47)
University or more	13.9 (0.75)	44.7 (2.30)	29.8 (2.18)

SE, standard error

Note: Current smoker defined as having ever smoked >100 cigarettes in lifetime and currently smoke every day or some days; former smoker defined as having ever smoked >100 cigarettes in lifetime but not currently smoking

* Prevalence of smoking is age-standardized

The prevalence of current smoking was 22.8% (SE, 2.84) among adults with physician-diagnosed heart disease, 26.8% (6.81) among those with diagnosed high blood pressure, 21.3% (2.46) among those with diagnosed high blood cholesterol and 20.5% (2.56) among those with diagnosed diabetes mellitus.

### Comment and note on tobacco legislation, control policies, programmes in Jordan

The well-known adverse effects of smoking and the documented benefits of quitting [13] notwithstanding, the prevalence of smoking among Jordanian youth and adults remains high. Smoking behavior among women may be higher than that reported here as women may deny their smoking behavior and/or underestimate their frequency of smoking. As a result of second-hand smoking, women's smoking exposure almost certainly exceeds that reflected in their own smoking behaviour. The prevalence of smoking among young and middle aged Jordanian men is similar to that of the US adult population during the late 1960s/early 1970s [14]. In Egypt the prevalence of lifetime smoking was 20% among boys and 5% among girls according to data from the 2005 GYTS while the prevalence among men (aged 15-65 years) was 34% according to data from the WHO's Global Infobase https://apps.who.int/infobase/report.aspx. Similarly high prevalences have been observed among boys (45% ever smoked, 25% currently smoke, 2006 GYTS) and men (20%-42% currently smoke, 2006/7 Iraq Family Health Survey; men aged 19-64 years) in Iraq.

The relatively lower prevalence rate of current smoking in patients with prevalent heart disease and heart disease risk factors in Jordan is easy to explain on the basis of patients quitting the habit after diagnosis with these conditions. In addition, poor survival of smokers suffering from heart disease and its risk factors may, in part, provide another explanation. Smoking cessation is essential for patients with CHD. However, current smoking remained unacceptably high in these patients. Current guidelines recommend that clinicians ask about tobacco use and provide counseling about quitting within the context of a comprehensive plan for secondary prevention [15,16]. Available strategies include identifying and documenting smoking status in all patients, referral for consultation and counseling, prescription of appropriate drugs in accordance with clinical guidelines, and the provision of quit lines and community support services [17]. In addition, initiatives to promote cessation at the work site are needed, as is enforcement of smoke-free legislation in schools and public places.

Jordan has an extensive history with tobacco control policies and programmes that have shaped its current tobacco control infrastructure. Jordan's initial anti-smoking regulation was part of a public health law issued in 1971. This initial legislation established jail sentences not exceed four months or fines (ranging from JD 25 to JD 500, or both penalties, [1 Jordanian Dinar (JD) = 1.41 US dollars]) but was challenged by the absence of enforcement mechanisms and application of penalties for those who smoked in public places and on public transport or promoted tobacco use through advertisements. In November 2001, legislation, included as part of Juvenile Monitoring Legislation, was put in place to restrict tobacco sales to minors with penalties for minors (e.g., a JD 20 fine for a first-time violation; fine doubled if the offence were to be repeated) and for the vendor (e.g., a JD 100 fine and a jail sentence of up to one year). In May 2003, Jordan adopted the Framework Convention on Tobacco Control (FCTC) with a tobacco control strategy that included a general ban on tobacco advertising, raising of public awareness on the hazards of tobacco use, enforcement of legislation, and encouragement of smoking cessation, among others. (*N.B*. The 2003 tobacco control country profile can be found online at http://www.who.int/tobacco/media/en/Jordan.pdf.) For example, a picture warning that covers 50% of the package size is now required on all cigarette packages in Jordan.

More recently (in 2008), Jordan's public health law was amended to prohibit smoking in public and private institutions and all public facilities including hospitals, healthcare centres, schools, cinemas, theatres, libraries, museums, public and nongovernmental buildings, public transport vehicles, airports, closed playgrounds, lecture halls and any other location at the discretion of the Minister of Health. Smoking is also prohibited inside Amman's shopping malls, and in addition to posted warning signs, the MOH has required five star restaurants in Amman to identify smoke-free places for non-smokers. Beginning June 2009, smoking was banned inside Amman's fast-food outlets. 'No smoking' signs were widely distributed and posted in every MOH facility, other major health facilities (hospitals, large health centres, etc), airports, and other venues. Penalties were established in this section in more formative way compared with prior legislation. In addition, the new legislation provided clear mechanisms for organizing the supervision and monitoring of the smoking ban. For example, the prohibition of smoking among staff in health facilities in the country was accompanied by a decision by the Minister of Health to penalize Ministry staff who smoked in health facilities both administratively and monetarily through reductions in wages and benefits. At the international airport, where smoking is now prohibited with the exception of designated smoking areas, focal points for monitoring adherence were also assigned. Effectiveness of these policies, however, remains to be measured.

In conclusion, while the current infrastructure for tobacco control is a beginning, opportunities remain to improve anti-smoking policies and programmes particularly in areas of enforcement. The prevalence of smoking in Jordan, particularly among men, remains unacceptably high, and opportunities exist to further reduce smoking among both youth and adults and particularly among patients with smoking-related diseases. Of course, it is hoped that the tobacco control policies will, in part, result in a reduction in smoking prevalence; however, such policies cannot work in isolation. Socio-cultural norms, whereby smoking among men is a common and accepted part of daily life with little or no societal perception of smoking as a negative behaviour, present a challenge to tobacco control. Ultimately, smokers must decide that they need to quit smoking. Smoking cessation programmes that offer free-of-charge counseling and nicotine replacement medication for those who wish to quit smoking as well as quit hotlines have been implemented in Jordan in the past, but their widespread use has not been sustained and some suggest that additional effort is needed to educate and counsel health professionals as well as provide them the necessary behavioral intervention skills for smoking cessation [18]. Effective tobacco-related awareness programmes, particularly anti-tobacco peer education programmes targeting youth, must be implemented more widely across the country. Combating tobacco use in Jordan will require partnerships and long-term commitments between both private and public institutions as well as within local communities.

## Competing interests

The authors declare that they have no competing interests.

## Authors' contributions

Study conception and design: AB, MAN, DWB. Acquisition of data: AB, MAN. Analysis and interpretation of data: MAN, DWB. Drafting of manuscript: AB, MAN, AB, DWB, HTW. Critical revision: AB, MAN, AB, DWB, HTW. All authors read and approved the final manuscript.
